# Electrocardiogram-Based Biometric Identification Using Mixed Feature Extraction and Sparse Representation

**DOI:** 10.3390/s23229179

**Published:** 2023-11-14

**Authors:** Xu Zhang, Qifeng Liu, Dong He, Hui Suo, Chun Zhao

**Affiliations:** 1 State Key Laboratory of Integrated Optoelectronics, College of Electronic Science and Engineering, Jilin University, Changchun 130015, China; xuzhang21@mails.jlu.edu.cn (X.Z.); suohui@jlu.edu.cn (H.S.); zchun@jlu.edu.cn (C.Z.); 2 School of Preparatory Education, Jilin University, Changchun 130015, China

**Keywords:** electrocardiogram (ECG), biometric, wavelet, sparse coding, dictionary learning

## Abstract

(1) Background: The ability to recognize identities is an essential component of security. Electrocardiogram (ECG) signals have gained popularity for identity recognition because of their universal, unique, stable, and measurable characteristics. To ensure accurate identification of ECG signals, this paper proposes an approach which involves mixed feature sampling, sparse representation, and recognition. (2) Methods: This paper introduces a new method of identifying individuals through their ECG signals. This technique combines the extraction of fixed ECG features and specific frequency features to improve accuracy in ECG identity recognition. This approach uses the wavelet transform to extract frequency bands which contain personal information features from the ECG signals. These bands are reconstructed, and the single R-peak localization determines the ECG window. The signals are segmented and standardized based on the located windows. A sparse dictionary is created using the standardized ECG signals, and the KSVD (K-Orthogonal Matching Pursuit) algorithm is employed to project ECG target signals into a sparse vector–matrix representation. To extract the final representation of the target signals for identification, the sparse coefficient vectors in the signals are maximally pooled. For recognition, the co-dimensional bundle search method is used in this paper. (3) Results: This paper utilizes the publicly available European ST-T database for our study. Specifically, this paper selects ECG signals from 20, 50 and 70 subjects, each with 30 testing segments. The method proposed in this paper achieved recognition rates of 99.14%, 99.09%, and 99.05%, respectively. (4) Conclusion: The experiments indicate that the method proposed in this paper can accurately capture, represent and identify ECG signals.

## 1. Introduction

ECG signals are unique to each individual and show consistent characteristics in various scenarios. They can be used to recognize individual identity over a long period and under different circumstances [1]. Additionally, it is easy to extract ECG signals, and with the increasing popularity of modern ECG devices, this technology has even greater potential [2]. After nearly 20 years of development, important research results have been achieved [3,4]. One popular research direction in biometrics involves the multimodal fusion of ECG signals with other biometric technologies, such as faces and fingerprints, to enhance the reliability and accuracy of identity recognition [5,6].

Accurate feature extraction is essential for ECG signal recognition, which can be achieved through reference point and non-reference point methods [7]. The reference point method involves extracting information, such as timing and amplitude, usually represented by P, Q, R, S, and T waves. This method of feature extraction can achieve relatively higher accuracy. Silva et al. extracted P waves, QRS complex, and T waves by detecting the peak points of R waves. The amplitudes and cycles of these waveforms were used as features, and the K-Nearest Neighbor (KNN) algorithm was used for identity recognition [8]. Pal et al. extracted features of P, R, Q, and T waves, using the KPCA algorithm and subsequently recognized them using Euclidean Distance [9]. Kiran et al. extracted six optimal P-QRS-T segments based on their priority and positions, and employed the SVM (Support Vector Machine) algorithm for classification [10]. Gurkan et al. extracted QRS complex and used convolutional neural networks for recognition [11].

The non-reference point method does not require extracting reference points from ECG signals. Instead, it uses concealed features from the waveforms for identification [12]. By reducing constraints on feature point extraction, this method demonstrated broader applicability, heightened recognition stability, and increased adaptability. Agrafioti et al. utilized the Discrete Cosine Transform (DCT) to extract features and reduce the dimensionality of ECG signals. They subsequently employed the KNN algorithm for identity recognition [13]. Plataniotis et al. proposed a non-reference point method for extracting ECG signal identity features using an AC (Auto Correlation)/DCT algorithm. The method involves the use of the DCT algorithm to extract ECG features, followed by feature recognition using the Gaussian Function and Euclidean Distance [14]. Coutinho et al. utilized the average single-beat waveform as the feature input. They employed data compression technology based on the Ziv Merhav cross-analysis algorithm to reduce the dimensionality of the features. These were then applied to 19 healthy individuals to verify the effectiveness of recognition [15]. Alotaiby et al. first decomposed the frequency band features of the signals, extracted statistical features from each designated frequency band to form a vector form, and input them into a random forest model for identity recognition [16].

In addition to the two types of detection methods mentioned above, the wavelet transform is also a commonly used method for feature extraction [17]. Wavelet transform is a powerful tool to analyze ECG signals by decomposing them into wavelet functions of different scales and times. It is often used for ECG signal processing tasks such as denoising, QRS detection, ST segment analysis, and T-wave analysis. Mohanty et al. adopted wavelet transform to denoise ECG signals, and then extracted the features of R peaks and the QRS complex. The classification accuracy achieved 98.17% in the MIT-BIH dataset [18]. Kumar et al. proposed a stationary wavelet transform algorithm for denoising ECG signals [19]. In comparison to other decomposition algorithms, this specific algorithm can preserve a greater number of components from ECG signals. Kumar’s report employed a wavelet multiscale transformation technique for ECG signal analysis. This approach captured different frequency components of the signals, along with their temporal changes.

All the methods mentioned above have their limitations. The accuracy of the reference point method depends on placing and calculating reference points precisely. However, during the collection of ECG signals, there can be various sources of noise interference, which can significantly affect the recognition accuracy due to imprecise reference point positioning [20]. Non-reference point method extracts redundant information, hindering real-time recognition and causing high computational costs [21]. The wavelet transform is a signal processing method which is very sensitive to noises in signal frequency. It can often identify high-frequency noise in ECG signals. However, when the signal-to-noise ratio is low, the wavelet transform might mistakenly identify and extract inaccurate frequency components, which can ultimately affect the accuracy of the ECG features [22,23].

Recently, a comprehensive feature extraction method has been proposed which combines reference and non-reference point methods to address the limitations of the single methods. The goal of this method is to fully utilize the advantages of reference point method without overly relying on their accuracy, while also extracting features through non-reference point method, thereby reducing the impact of the aforementioned issues to a certain extent. D. Melter et al. used the Pan–Tompkins (P-T) algorithm to detect the R peaks of the QRS complex and extract non-fiducial features based on (AC/DCT). This method has yielded outstanding results which have provided us with a pioneering insight. This paper used a hybrid approach to extract signal features through a combination of the P-T algorithm and KSVD algorithm to extract features, and then apply the co-dimensional bundle search method proposed in this paper for recognition purposes. The major contributions of our study are as follows:

- This study aims to decompose signals in terms of their frequencies, and extract frequency components which exhibit significant feature differences for recognition purposes. The ECG signal features obtained through this recognition method are not specific feature components, but rather a larger and unique set of ECG components in the signals. Although this set cannot represent the entire ECG signals, it covers the most distinct portion. Subsequently, this paper utilizes the KSVD algorithm for sparse processing. This approach overcomes the limitations of the previous methods and generates matrices which have no specific meaning but have significant specificity.
- This study researches sparse matrices and develops a recognition algorithm suitable for this study. This study collected a large number of feature matrices and constructed a matrix set specifically for ECG signals. Within these matrices, we performed mathematical operations on the feature components which are in the same position, and the final result was represented as the distance. We compared the signals that need to be identified with the matrices in the set to obtain a new set that includes various distance values. By assigning different weights to different distances and performing corresponding calculations, we obtained the eigenvalues. Finally, we were able to determine the recognition results by analyzing the relationship between the threshold and feature values.

Further, this paper is organized as follows. Section 2 describes the database and ECG-related algorithms used in this paper. Section 3 introduces the key components of the method proposed in this paper. Section 4 describes the experimental results and concluding remarks. Section 5 presents the conclusion and future prospects.

## 2. Related Work

### 2.1. Database

The European ST-T database contains two-channel ECG recordings from 79 subjects, with each recording lasting two hours. The recordings were sampled at 250 Hz with a 12 bit resolution and included leads I (MLI) and II (MLII) for each subject. The data were uniformly stored in 212 data storage modes [24]. This is a publicly accessible database created by the European Heart Association to evaluate the effectiveness of algorithms used for detecting ST segments and T-wave abnormalities in ECG signals. The subjects included in this database are either diagnosed with or suspected of having heart-related conditions. This study uses 120,000 data points (about 7–8 min) which are divided into two sets: 80,000 points (5–6 min) for dictionary training, and 40,000 points (2–3 min) for validation purposes. For the training and validation sets, 1200 data points (3–6 s) are used as the standard units for data processing.

### 2.2. Wavelet Transform

The main purpose of wavelet multiscale transformation is to explore the properties of wavelet functions and scale functions. Among the various wavelet functions, the Daubechies wavelet (db wavelet) is widely used due to its excellent noise reduction capabilities. Consequently, it is a popular choice for extracting features and reducing noise in ECG signals [25,26]. In the context of scale functions, wavelet multiscale transformation decomposes signals at various scales. For a given wavelet function ψ(t), the wavelet transform of $f(t)\in L^{2}(R)$ is:

(1) $$W_{f}(a,b)={\left|a\right|}^{-\frac{1}{2}}\int_{-\infty }^{\infty }f(t)*\bar{\psi }(\frac{t-b}{a})dt$$

where ***a*** represents the scale factor that corresponds to the frequency domain characteristics; ***b*** represents the time factor reflecting the time-domain characteristics. The wavelet transform coefficient, denoted as $W_{f}(a,b)$, is the inner product of the wavelet signal at scale ***a*** and displacement ***b***, indicating the similarity between the signals and the wavelet represented by that point. The ECG signals are extracted through multi-level low-pass and high-pass filters to obtain their features. ECG signals are decomposed into frequency components of different scales.

The reconstruction process may introduce signal distortion and affects denoising effectiveness at high decomposition levels in the wavelet multiscale transform [27]. It is important to note that the signal’s sensitivity is affected by the parameters chosen for analysis. During the wavelet transformation, certain high or low-frequency components are discarded, leading to signal compression and decomposition. However, this process can lead to the loss of significant information, and important signal features may be either blurred or completely discarded.

### 2.3. R-Peak Detection

**Figure 1:**
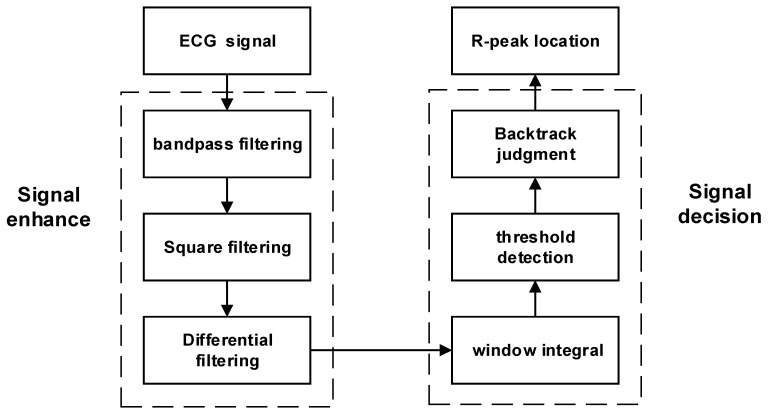
Algorithm flow for R-peak localization.

When analyzing ECG signals, the R peak is the most prominent feature and is relatively simple to identify. We applied the P-T algorithm and made some improvements on this basis to obtain R peaks of ECG signals. We adopted an adaptive QRS waveform detection algorithm using two thresholds to improve accuracy. By using the R peak as a reference, multiple signals can be segmented more accurately [28,29]. Figure 1 shows the steps involved in the algorithm process. First, the ECG signals are filtered and processed to improve the R waves. Then, a differential operation is performed on the filtered signals, which highlights the quickly changing part of the QRS complex. By using sliding integration, the position of the R peak is determined. To filter the peaks, two threshold conditions are set, where the second threshold is half the value of the first threshold. If a peak is higher than the first threshold, it is considered to be an R wave. If it is lower than the first threshold, it is classified as noise.

It is important to note that there are limitations to this method. The differences in how adaptive algorithms are implemented and the settings of their parameters can lead to inconsistent outcomes among various implementations. The absence of standardized implementation and evaluation could potentially undermine the reliability of these algorithms. Additionally, when ECG signals are subjected to substantial interference or noise, the algorithm might yield false positives or missed detections, failing to identify specific R peaks, and thereby diminishing detection accuracy.

### 2.4. Sparse Representation

Managing the large amount of ECG signal data requires reducing the sample dimensionality. Sparse representation can depict signals with fewer non-zero coefficients, which helps create a compressed signal representation. This method is stable and robust, even when faced with noise or interference. The sparse representation of the signals maintains a commendable level of sparsity, even in such situations [30]. The concept of a dictionary lies within the realm of sparse expression. Composed of basic atoms, the dictionary can accurately represent complex signals by combining different atoms. To acquire a dictionary, it is necessary to construct it from a signal learning perspective. This approach can be applied to any type of signal and has the potential to produce sparse depictions that are notably accurate.

Dictionary construction and training are two key steps to achieve sparsity when using the dictionary method. In this study, a comprehensive initialization dictionary was constructed and trained with the KSVD algorithm [31,32]. The dictionary refinement process involves using the OMP (Orthogonal Matching Pursuit) tracking algorithm to update the dictionary [33]. The KSVD algorithm has two distinct stages. First, the OMP algorithm is applied to achieve a sparse representation of the data and obtain the coefficient matrix X. This matrix corresponds to the dictionary. Second, using the obtained coefficient matrix X, the KSVD algorithm updates the dictionary column by column, resulting in an improved dictionary. At the same time, the coefficient matrix is also updated. This iterative process eventually yields a fully refined dictionary.

The KSVD algorithm can be computationally intensive due to high-complexity operations such as Singular Value Decomposition (SVD) required in each iteration. Additionally, it is sensitive to the initial dictionary, which can result in various local optimal solutions. This sensitivity affects both the algorithm performance and stability. KSVD is commonly used for tasks like image compression and denoising. In the new approach, this paper combines one-dimensional ECG signals into small batches, creating two-dimensional signals. These signals are treated as miniature images in the second dimension, which effectively addresses the computational complexity issue. 

## 3. Methodology of the Proposed Work Methods

**Figure 2:**
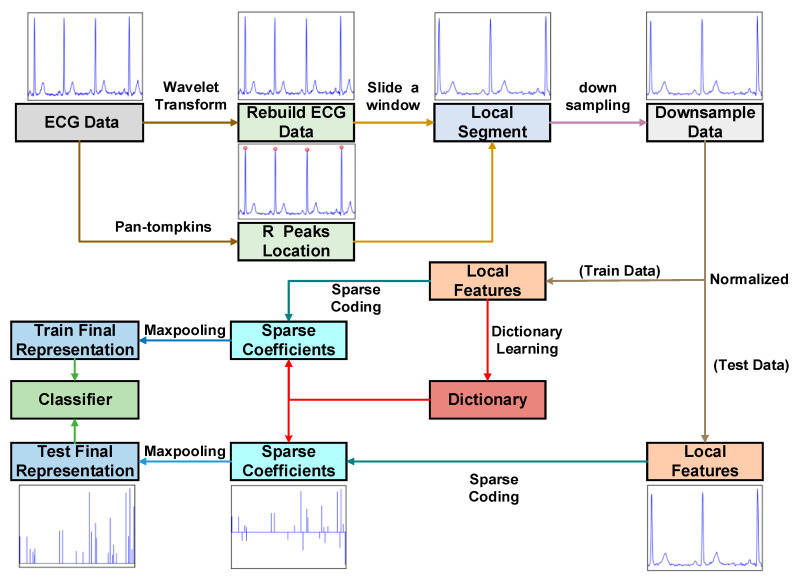
The framework diagram of ECG signal recognition.

This paper proposes a new approach that combines mixed feature extraction with data dimensionality reduction using Sparse Dictionary. This paper also addresses the issue of solving sparse matrices by employing the co-dimensional bundle search technique. This study utilizes the following method to implement the algorithm flow, which includes four main modules: data preprocessing, feature extraction, Sparse Dictionary and classification. To summarize the proposed method, the following steps are taken (Figure 2 and Table 1):

### 3.1. Data Preprocessing

Wavelet signal processing involves various steps, including sampling, decomposition, signal processing, and reconstruction. For sampling, the Nyquist theorem is applied, where the sampling rate should be at least two times larger than the signal detailed frequency to prevent distortion. Then, the signal is decomposed into its approximate component and detailed component, and the approximate component is further decomposed into an approximate component and a detailed component. This process is known as multiscale decomposition. This paper denoises a signal and discretizes it into $2^{n}$ sampling points.

(2) akn=fk2n 0<k<2n−1

Perform downsampling on the original signal by discarding its odd parts and applying a filter to calculate the downsampling sequence, ***D***. The filters ***h*** and ***l*** correspond to high-pass and low-pass filters, respectively.

(3) $$a^{j-1}=D\left(l\;\times \;a^{j}\right),\;\;b^{j-1}=D(h{\;\times \;a}^{j})$$

$a^{j-1}$ represents the approximation component and $b^{j-1}$ represents the detailed component.

**Figure 3:**
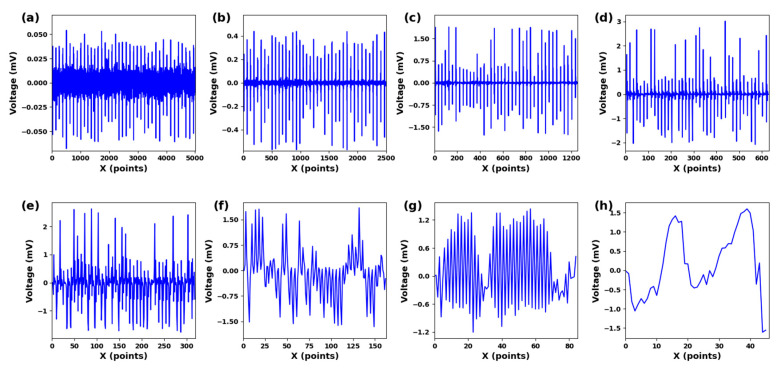
(**a**) cD1. (**b**) cD2. (**c**) cD3. (**d**) cD4. (**e**) cD5. (**f**) cD6. (**g**) cD7. (**h**) cD8. Different scale components under 8-scale decomposition of ECG signals.

The ECG signal has an amplitude range of 10 uV to 4 mV, with the typical value being 1 mV. The frequency range spans from 0.05 to 100 Hz, and 90% of the QRS spectrum energy of ECG falls between 0.25 and 45 Hz [34]. Significant feature components are extracted by suppressing noise and unclear features. This paper uses the db4 wavelet basis with good correlation to the signal and perform 8-layer decomposition. Figure 3a–h represents the components from low frequency to high frequency at 0–100 Hz. The QRS frequency component is mainly included in Figure 3a–h. Frequency components within this range can improve the recognition performance of QRS wave frequency bands.

This paper reconstructs the processed approximate coefficients and wavelet coefficients by using a discrete filter implementation form.

(4) $$a_{k}^{j}=\sum {\bar{l}}_{\left(k-2\right)}a_{l}^{j-1}+\sum {\bar{h}}_{\left(k-2\right)}b_{l}^{j-1}$$

By using $a_{k}^{j-1}$ upsampling to uniformly distribute 0 in a sequence, a new sequence is created with 0 at every odd position. Each non-zero term of the original sequence now has an even index, resulting in a new sequence $U_{X}$. The reconstructed high-pass filter and reconstructed low-pass filter are used by applying discrete filters $\bar{h}$ and $\bar{l}$, respectively. The convolution form is:

(5) $$a_{k}^{j}=\bar{l}*\left(U\left(a^{j-1}\right)\right)+\bar{h}*\left(U\left(a^{j-1}\right)\right)$$

After selecting the appropriate wavelet function and decomposition scale, we can decompose and process the signals to obtain the desired reconstructed ECG signals.

### 3.2. Feature Extraction

The adaptive dual-threshold QRS wave detection algorithm is a reliable method to accurately identify the most prominent R peaks within an ECG signal. This process of detecting R peaks helps to precisely locate them, which in turn facilitates the segmentation of the ECG signals into distinct segments.

**Figure 4:**
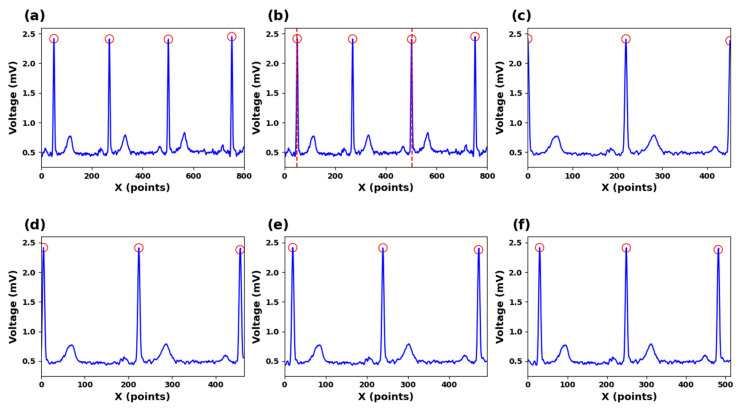
(**a**) ECG signals for R-peak detection. (**b**) Segmented ECG signals. (**c**) Zero-point expanded ECG signals. (**d**) Ten-points expanded ECG signals (**e**) Twenty-points expanded ECG signals (**f**) Thirty-points expanded ECG signals.

As depicted in Figure 4a–c, the position of the R peak is used as a reference point, three consecutive R peaks are selected, and an R-R-R sequence is created which contains two complete ECG cycles. In Figure 4d–g, a complete waveform of the edge R peak within this sequence is captured. By extracting segmented signal portions from this sequence, signal continuity is maintained throughout the process. Localized segments with consistent dimensions are derived from segmented signal portions following an L2 regularization procedure, and these localized segments are used to train the dictionary and for recognition tasks. 

In this study, the segmentation points are determined by using two R-wave points separated by one R wave. The purpose is to ensure that each segmented ECG signal segment covers a complete cardiac cycle. However, using R-wave points as segmentation lines for input signals resulted in a relatively low recognition rate. The reason could be that the R-wave features at both ends of the signals are not fully represented, which affects the accuracy of the recognition. Our findings indicate that, as both ends expand outward, the accuracy gradually increases. When the feature values of the R peaks on both sides are included in the signals, the accuracy of recognition tends to be stable.

### 3.3. Sparse Dictionary

In Step 3, the KSVD algorithm is used to obtain the sparse representation of the ECG signals. Firstly, an over-complete dictionary is given, $D\in M^{d\times K}$ (***K > d***). The problem of sparse representation involves finding a linear combination of a small number of basic elements from the dictionary to accurately represent a signal $x\in M^{d}$. ***x*** is the sparse coefficient vector that specifies the projection weight. The signals decompose into basic combinations of atoms in the dictionary. The $l_{0}$ norm can be approximated using the $l_{1}$ norm if the solution is sparse enough, i.e.,

(6) $$\hat{\alpha }=argmin{\left\|\alpha \right\|}_{1}\;\;s.t.D\alpha =x.$$

In sparse representation schemes, two issues need to be addressed. The primary issue is how to obtain information from training data, while the second issue involves how to use the learned dictionary to calculate the generated signals. Consider a set of local segments $X=[x_{1},x_{2},\ldots ,x_{N}]\in M^{d\times K}$, the dictionary can be taught by optimizing an empirical cost function:

(7) $${min\left\|Y-DX\right\|}_{F}^{2}+\lambda {\left\|x_{i}\right\|}_{1}$$

The dictionary remains unchanged. The OMP algorithm is employed to compute the sparse coding matrix ***X***, as outlined in Equation (7) [32]. Once ***X*** is obtained, the process involves incremental updates to the dictionary on a column-by-column basis. The ***k***-column of the dictionary is updated, where $d_{k}$ represents the ***k***-column vector of the dictionary ***D***, and $x_{T}^{k}$ signifies the ***k***-row vector of the sparse matrix **X**. This procedure applies specifically to the initial phase of the model.

(8) $${\left\|Y-DX\right\|}_{F}^{2}={\left\|E_{k}-d_{k}x_{T}^{k}\right\|}_{F}^{2}$$

The residual can be described by Equation (9):

(9) $$E_{k}=Y-\sum_{j\neq k}d_{j}x_{T}^{j}$$

And then, the optimization problem can be transformed into:

(10) $${min\left\|Y-DX\right\|}_{F}^{2}+\lambda {\left\|x_{i}\right\|}_{1}$$

By using singular-value decomposition, update each column of atoms and each row of sparse encoding to find the optimal $d_{k}$ and $x_{T}^{k}$. Complete the update of the dictionary.

**Figure 5:**
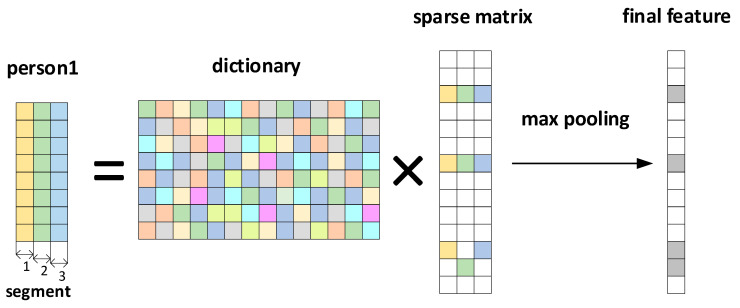
Sparse representation and max pooling process.

**Figure 6:**
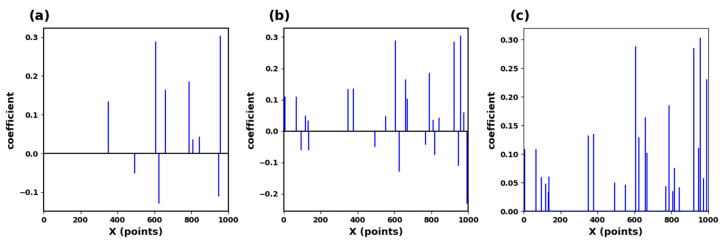
(**a**) Sparse matrix of ECG signals. (**b**) Sparse matrix collection of ECG signals. (**c**) Max pooling of sparse feature coefficients.

The schematic process is shown in Figure 5. This study requires the dictionary structure to contain 1000 atoms, a maximum number of iterations of 300, a tolerance of 1.0 × 10^−6^ for sparsity in presentation results, and a sparsity of 10. This means that the number of data points in each local segment of the input ECG is reduced from the original 400 points to 10. We input a signal with the length of 400, and the situation obtained by the KSVD algorithm is shown in Figure 6a. After inputting three such data segments for sparse representation and superposition, we generate Figure 6b. Figure 6c is the modulus of Figure 6b. We input multiple data segments of the subjects’ ECG signals and obtain a sparse matrix through the Sparse Dictionary. 

Assuming that ***N*** segment signals undergo sparsity to obtain a sparse matrix: ***A =* [**${[\alpha }_{1},\alpha_{2},\ldots ,\alpha_{M}..]\in R^{K\times N}$], maxpooling is the maximum absolute value of the matrix ***A*** at the same position of each sparse segment [35]. Then, the matrix data are obtained:

(11) $$\beta =max\left\{\left|\alpha_{1k}\right|,\left|\alpha_{2k}\right|,...,\left|\alpha_{Nk}\right|\right\}$$

where $\beta_{k}$ is the ***k***-th element of β, and $\alpha_{ik}$ is the sparse coefficient at the ***k*-th** position of the ***i*** input segment. This study selected 70 subjects for training, totaling 4900 segments. A segment of data is evenly divided into three segments to generate a ***D*** × 3 matrix. After sparse representation, a ***K*** × 3 matrix is obtained. Then, the maximum pool operation is performed on each column to obtain a K × 1, and then take the modular value of the characteristics in the matrix ***K*** × 1. The result is shown in Figure 6c.

### 3.4. Classification

In Step 4, an individual identity recognition matrix is formulated. The individual identity recognition features, obtained by processing the ECG signals of the subjects to be identified, are then traversed within the individual identity recognition matrix using the co-dimensional bundle search method. This ultimately achieved successful ECG identification.

The process of final signal recognition is shown in Table 2: The input ECG signals are preprocessed and sparsely represented, and finally become a sparse matrix (Final Feature) which is sparse enough. Most elements in the sparse matrix are zero (without features), and only a small number of elements have features (values are not zero). This study proposes a method for identity recognition using bundle search based on this work. Firstly, a standard set is established. As shown in Equation (12), for each subject, a representative matrix is selected from multiple trained sparse matrices, and it is used as a record of the subject in the standard set. Whenever a new subject inputs $\beta^{t}$***(1 × N)***, the sparse matrix will be calculated in the standard set $\beta^{c}\left(M\times N\right)$ to determine the identity of the subject. The specific calculation method is as follows:

First, traverse the non-zero features in the input matrix to form the matrix $\beta^{t1}$***= {***$\beta^{1}$***,***$\beta^{2}$***, …,*** $\beta^{k}$**}**. Then, based on the input data traversal, take the corresponding matrix at the corresponding position in the standard set:

(12) $$\beta^{t1}=\left\{\begin{array}{cccc} \beta_{1,1} & \beta_{1,2} & \cdots & \beta_{1,k}\\ \beta_{2,1} & \beta_{2,2} & \cdots & \beta_{2,k}\\ \beta_{3,1} & \beta_{3,2} & \cdots & \beta_{3,k}\\ \cdots & \cdots & \cdots & \cdots \\ \beta_{m,1} & \beta_{m,2} & \cdots & \beta_{m,k} \end{array}\right\}$$

For each feature, find the corresponding feature in the standard set and calculate the distance ***d*** from it. Traverse all features to obtain a set of distance $d_{l}$. Sort these distances to obtain matrix ***L***. Determine the attribution of the features based on the nearest local feature set in matrix ***L***. Traverse all features. Finally, based on the matrix with the most feature attribution, identify the subject to which the feature matrix group belongs.

Each feature $\beta^{i}\;$ finds the corresponding feature in the standard set and calculates the distance as:

(13) $$d_{i,m}=|\beta^{i}-\beta_{z,i}|(z=1,2,\ldots m)$$

Traverse all features to obtain a set of distances $d_{i,total}$; sort these distances to obtain a list ***L*** = $argmin(d_{i,total})$; and determine the subject to which the feature belongs based on the closest subject in list ***L***. Traverse all features. Finally, identify the subject who matches the most features. Additionally, design a threshold $R_{t}$. Add the set of distances, and, if the total distance is greater than this threshold, it is determined that the ECG signals do not belong to the subject in the dataset. Finally, under the European ST-T dataset, 20, 50, and 70 subjects’ ECG signals were selected, each with 30 test segments. The recognition rates of 99.14%, 99.09%, and 99.05% were achieved, respectively, using the method proposed in this paper, indicating that the recognition method proposed in this paper can effectively perform ECG identity recognition.

## 4. Discussion

### 4.1. The Impact of Extraction Methods on Accuracy

**Figure 7:**
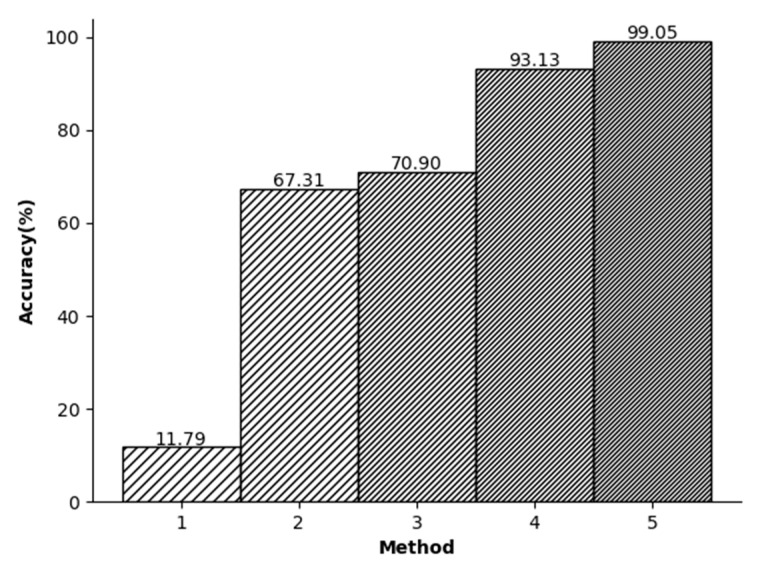
(1) R-point detection (2) Random split+KSVD (3) Random split+Wavelet+KSVD (4) R split+KSVD (5) R split + Wavelet + KSVD.

In this study, a series of experimental comparisons were conducted to evaluate the accuracy of ECG signal recognition. Figure 7 shows that when only the R-peak amplitude of the ECG signals was used for recognition, the accuracy was found to be relatively low. However, when the KSVD algorithm for sparse representation was employed, the accuracy increased significantly. Despite the utilization of random signal segments and the integration of wavelet transform and KSVD algorithm to compress the signals, the accuracy was still unsatisfactory. This limitation can be attributed to the selection of random segments. Randomly segmenting the signals without a reference point can lead to the randomness of the position of the signal components. In this case, the matrix components represented through sparse representation also exhibit randomness. This leads to lower accuracy. In the context of ECG signals, segments with the same length show diverse signal magnitudes, leading to relatively randomized distributions of their components. This randomness contributed to the challenge of achieving a sufficiently high recognition rate.

In the third experiment, shown in Figure 7, initially the R peak is used to segment the signals. Then, a downsampling method is used to reduce the dimensionality of the data. In general, in the database we used, the length of the R-R-R data segment is usually slightly different and usually slightly larger than the dimension we adopt. Therefore, the purpose of this step is to ensure that each signal contains approximately the same amount of information. Finally, the wavelet transform is employed to denoise the signals and enables the specialized-feature extraction. As a result, this study achieved an impressive accuracy rate of 99.05%.

The relationship between different experimental conditions and accuracy.

### 4.2. The Influence of ECG Signal Length and Denoising on Accuracy

**Figure 8:**
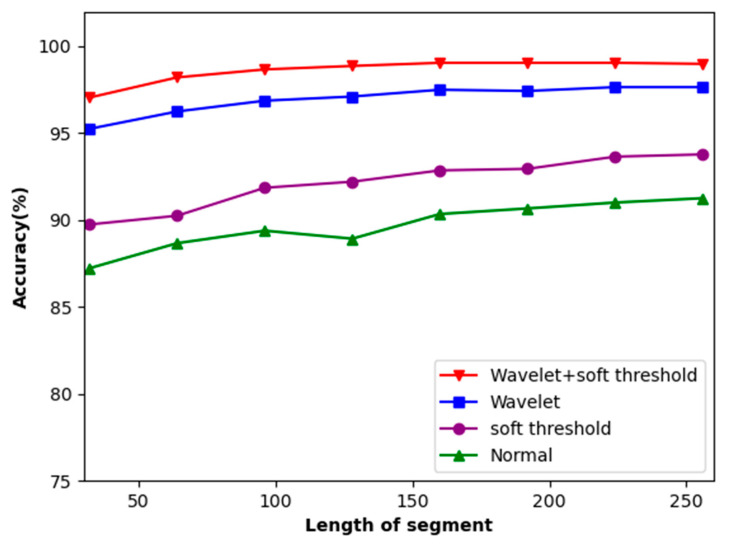
Accuracy of the dataset concerning the length of local segments.

The experimenters selected input lengths of 32, 64, 96, 128, 160, 192, 224, and 256 based on the actual test conditions. The experimental results are presented in Figure 8, depicting the outcomes of these input lengths. The accuracy of ECG recognition improves as the length of the intercepted ECG signal increases. Upon reaching the length of 160, the increase in accuracy stabilizes.

This method tackles the issue of noise in ECG signals. It identifies the original ECG signals and performs soft threshold denoising, followed by the wavelet transform. The signals before and after the addition of soft threshold denoising to wavelet transform are compared. As depicted in Figure 8, this method, combining both soft threshold denoising methods and the wavelet transform, achieved an 8% improvement in accuracy.

**Figure 9:**
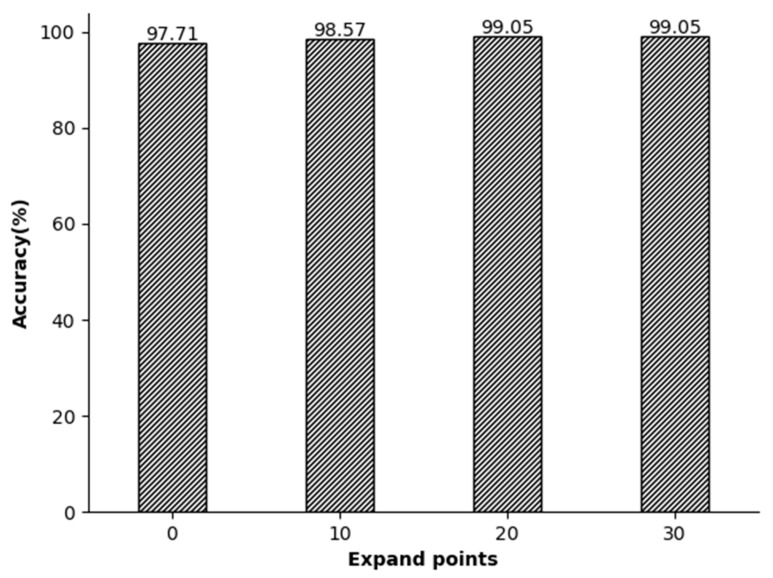
The relationship between the local segment length and accuracy.

In our analysis, the impact of segmented segments on accuracy was also evaluated. The segmentation point in the middle comprises two R-peak points separated by an R peak, and each ECG signal segment can capture an entire electrocardiogram cycle. The R-R-R sequence was expanded by 0, 10, 20, and 30 points both before and after the peaks. The experiments showed that using R peaks as segmentation lines on both ends resulted in lower recognition rates. This is because the signals of the R peaks on both sides are not complete as they are used as the segmentation points. Therefore, by continuously expanding the signal length, the experimenters ensure that the R peaks on both sides are completely contained in the information segments. After the expansion experiments of 10, 20, and 30 signal points, we found that the set of 20 points can perfectly restore the signals of the R peaks on both sides, making them complete and without redundant information (Figure 9). 

### 4.3. The Impact of Dictionary Length and Number of Iterations on Accuracy

**Figure 10:**
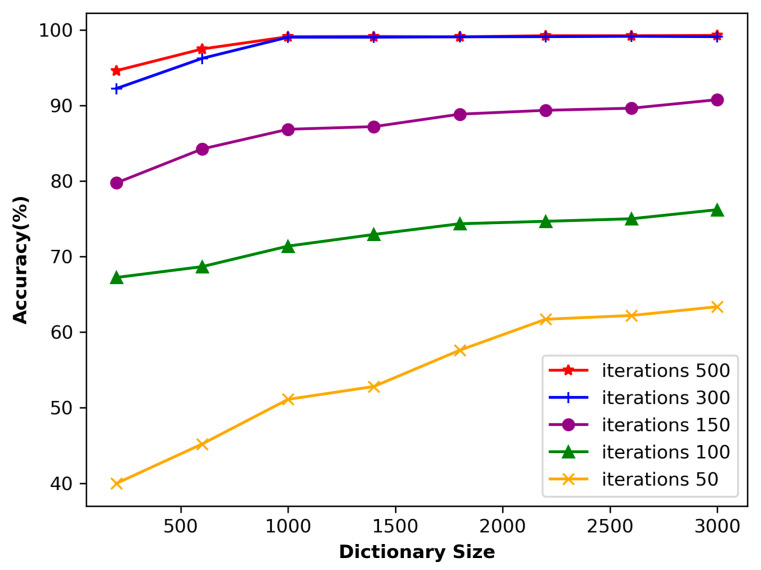
Accuracy of the dataset concerning the dictionary size.

Dictionary size represents the capacity or size of a dictionary, which represents the number of atoms contained in the dictionary. It determines the feature expression ability of the dictionary. A larger dictionary usually contains more features, which can better capture the details and complexity of input data. In this study, the ECG length segmentation was fixed at 160, and the dictionary size varied between 400 and 2000, with a step size of 400. Figure 10 shows the classification accuracy relative to the dictionary size. Generally speaking, the larger the dictionary, the higher the accuracy. The figure illustrates that an accuracy of 99.52% is achieved by using a dictionary size of 3000. However, the training time of a dictionary size of 3000 is dozens of times longer than that of the length of 1000. Therefore, this paper selects atoms with the length of 1000 to construct the dictionary.

If the dictionary is too large, it may lead to a significant increase in computational complexity. Usually, when the algorithm dictionary size adopted in this paper is doubled, the computational complexity will increase by 4–8 times. On the other hand, if the dictionary is too small, the noise in the final representation may have a greater impact on the results. In this study, the ECG length segmentation was fixed at 160, and the dictionary size varied from 400 to 2000, with a step size of 400. Figure 10 shows the classification accuracy related to the dictionary size. 

The number of input signal iterations has a significant impact on the learning ability of the dictionary. Figure 10 shows that when the number of iterations is only 50, the accuracy is relatively low despite the size of the dictionary. This is because the number of iterations is too small, which only allows the dictionary to capture a portion of the signal features, thereby affecting the learning result. As a result, using sparse representation to process input signals may lead to significant errors. However, as the number of iterations increases, the accuracy gradually improves. When the number of iterations reaches 300, the impact on accuracy gradually decreases. At this stage, it can be inferred that the dictionary is capable of effectively extracting most of the signal features.

### 4.4. Comparison with Other Articles

Based on the findings presented in Table 3, it is evident that the method proposed in this paper achieves a significant and consistent recognition rate. This can be attributed to the integration of a greater number of feature points in this study. The approach involves the adoption of R-peak extraction methods, data segmentation, extraction of the main components of QRS, and a dimensionality reduction in data segments. Compared with the QRS wave recognition method, this paper uses fewer features in recognition, significantly reducing the computational complexity of the classifier. Furthermore, when compared to ordinary single-feature recognition, this paper has a higher recognition rate. In addition, compared to the 3D VCG construction method in the table, our method has lower computational complexity. When the accuracy is similar, our method is easier to deploy, and therefore more suitable for mobile and portable applications. However, compared to some feature extraction methods mentioned in this paper, such as AC/DCT and FDM (Finite Difference Method), our method has a higher computational complexity. However, our method is suitable for tasks, but it requires high-quality sparse representation and has higher accuracy. In summary, compared to existing methods, the recognition method proposed in this paper has significant advantages.

## 5. Conclusions

A novel method for individual identification using ECG signals is proposed in this paper. The proposed approach starts with the wavelet transformation of the ECG signals, which extracts components that exhibit distinct QRS complex frequencies with a high concentration. Next, the ECG signals are reconstructed to facilitate denoising and feature enhancement processes. R peaks are located to extract feature segments, and a sparse representation is performed based on localized fragments. Recognition is achieved using our proposed co-dimension bundle search algorithm. We tested this method on the ST-T public database with 70 subjects and achieved an accuracy of 99.05%. Our research has shown promising results, and relies heavily on well-trained dictionaries. However, insufficient training leads to reduced accuracy, and our current dictionary may not run as expected on different datasets. Therefore, our future research will prioritize developing a more versatile dictionary that excels across various datasets with minimal training data.

## Figures and Tables

**Table 1:** ECG-based biometric identification using mixed feature extraction and sparse representation.

Step 1: Data preprocessing: Processing original data using wavelet transform; Reconstructing ECG signals.
Step 2: Feature extraction: Positioning R peaks; Splitting data into dual-cycle ECG signals; Downsampling and normalization of signals.
Step 3: Sparse Dictionary: Initializing dictionary D and updating the dictionary using the OMP algorithm; Determining the sparse coefficient and iteration number to obtain the coefficient matrix of signals.
Step 4: Classification: Using the co-dimensional bundle search for classification.

**Table 2:** Co-dimensional bundle search recognition.

Step 1: Establish a standard set 1. The matrix shown in Figure 6c as an element of a standard set. 2. Each subject takes 30 known matrices to establish a standard set.
Step 2: Calculate distance
1. Obtain an unknown sparse matrix.
2. Traverse the position of each non-zero element in the matrix and calculate the distance between each position and the same position as other elements in the dataset. Step 3: Assign tags and determine signal ownership
1. Sort the distances between the same positions in the matrix and find the subject represented by the nearest label. Affix the same label to it. 2. Obtain all the labels obtained from this matrix and sort them. 3. Make it belong to the one with the most tags.
4. Design a threshold R. 5. Calculate the average distance ***d*** between the matrix and all matrices in its label set. 6. If ***d*** < ***R***, the signals belong to this subject, otherwise the subject to whom the signal belongs is not in this set.

**Table 3:** Methods for measuring the biological characteristics of ECG signals (NS—Numbers of Subjects).

Author	Preparation	Decision	Dataset	NS	Results
This paper	R-R segmentation, DWT, KSVD	Same dimension beam search (this paper)	EU-ST-T	20 50 70	99.14% 99.09% 99.05%
Lee et al. [36]	R and T detection, R segmentation, resampling	Cosine, Euclidean, Manhattan dists., and CC	Private	55	93.30%
Dong et. [37]	Construction of 3D VCG with 12-lead ECG	Minimum L1 norm of the bank of errors	PTB	14 99	98.30% 93.30%
Pal et al. [38]	DWT fiducial det, P-QRS-T segmentation	Euclidean distance	PTB	100	97.10%
Dar et al. [39]	Local-Max R det, QRS segmentation	KNN	MIT ECG-ID	47 90	93.1% 83.2%
David et al. [40]	Pan–Tompkins, AC/DCT	A Clustering Algorithm	MIT	549	98.6%
Kim et al. [41]	amplitude, angle, Wavelet,	LSTM+ DRNN	MIT-BIH	47	99.8%
Binish et al. [42]	FDM + PT	RF+ ESD +SVM	BIH	50	97.9%

## Data Availability

The European ST-T dataset, which was generated by European Society of Cardiology (http://www.escardio.org/Pages/index.aspx).

## References

[1] Benouis M., Mostefai L., Costen N., Regouid M. (2021). ECG based biometric identification using one-dimensional local difference pattern. Biomed. Signal Process. Control.

[2] Biel L., Pettersson O., Philipson L., Wide P. (2001). ECG analysis: A new approach in human identification. IEEE Trans. Instrum. Meas..

[3] Uwaechia A.N., Ramli D.A. (2021). A Comprehensive Survey on ECG Signals as New Biometric Modality for Human Authentication: Recent Advances and Future Challenges. IEEE Access.

[4] Berkaya S.K., Uysal A.K., Gunal E.S., Ergin S., Gunal S., Gulmezoglu M.B. (2018). A survey on ECG analysis. Biomed. Signal Process. Control.

[5] Melzi P., Tolosana R., Vera-Rodriguez R. (2023). ECG Biometric Recognition: Review, System Proposal, and Benchmark Evaluation. IEEE Access.

[6] Ali M.M., Yannawar P., Gaikwad A. Study of edge detection methods based on palmprint lines. Proceedings of the 2016 International Conference on Electrical, Electronics, and Optimization Techniques (ICEEOT).

[7] Phukpattaranont P. (2015). QRS detection algorithm based on the quadratic filter. Expert Syst. Appl..

[8] Silva H., Gamboa H., Fred A. (2007). Applicability of lead v2 ECG measurements in biometrics. Med-E-Tel Proc..

[9] Pal A., Singh Y.N. (2018). ECG Biometric Recognition. Mathematics and Computing.

[10] Patro K.K., Kumar P.R. (2017). AMachine Learning Classification Approaches for Biometric Recognition System using ECG Signals. J. Eng. Sci. Technol. Rev..

[11] Gurkan H., Hanilci A. (2020). ECG based biometric identification method using QRS images and convolutional neural network. Pamukkale Univ. J. Eng. Sci..

[12] Choi G.-H., Bak E.-S., Pan S.-B. (2019). User identification system using 2D resized spectrogram features of ECG. IEEE Access.

[13] Agrafioti F., Hatzinakos D. ECG Based Recognition Using Second Order Statistics. Proceedings of the 6th Annual Communication Networks and Services Research Conference (CNSR 2008).

[14] Plataniotis K.N., Hatzinakos D., Lee J.K. ECG biometric recognition without fiducial detection. Proceedings of the 2006 Biometrics Symposium: Special Session on Research at the Biometric Consortium Conference.

[15] Coutinho D.P., Fred A.L.N., Figueiredo M.A.T. One-Lead ECG-based Personal Identification Using Ziv-Merhav Cross Parsing. Proceedings of the 2010 20th International Conference on Pattern Recognition.

[16] Alotaiby T.N., Alrshoud S.R., Alshebeili S.A., Aljafar L.M. (2019). ECG-Based Subject Identification Using Statistical Features and Random Forest. J. Sens..

[17] Seena V., Yomas J. A review on feature extraction and denoising of ECG signal using wavelet transform. Proceedings of the 2014 2nd International Conference on Devices, Circuits and Systems (ICDCS).

[18] Mohanty M., Biswal P.K., Sabut S.K. Feature Extraction of ECG signal for Detection of Ventricular Fibrillation. Proceedings of the Proceedings 2015 International Conference on Man and Machine Interfacing (MAMI).

[19] Kumar A., Tomar H., Mehla V.K., Komaragiri R., Kumar M. (2021). Stationary wavelet transform based ECG signal denoising method. ISA Trans..

[20] Al Alkeem E., Kim S.-K., Yeun C.Y., Zemerly M.J., Poon K.F., Gianini G., Yoo P.D. (2019). An enhanced electrocardiogram biometric authentication system using machine learning. IEEE Access.

[21] Mumtaz W., Rasheed S., Irfan A. (2021). Review of challenges associated with the EEG artifact removal methods. Biomed. Signal Process. Control.

[22] Singh B., Singh P., Budhiraja S. Various approaches to minimise noises in ECG signal: A survey. Proceedings of the 2015 Fifth International Conference on Advanced Computing & Communication Technologies.

[23] Sang Y.-F. (2013). A review on the applications of wavelet transform in hydrology time series analysis. Atmos. Res..

[24] Taddei A., Distante G., Emdin M., Pisani P., Moody G.B., Zeelenberg C., Marchesi C. (1992). The European ST-T database: Standard for evaluating systems for the analysis of ST-T changes in ambulatory electrocardiography. Eur. Heart J..

[25] Singh B.N., Tiwari A.K. (2006). Optimal selection of wavelet basis function applied to ECG signal denoising. Digit. Signal Process..

[26] Yadav S.K., Sinha R., Bora P.K. (2015). Electrocardiogram signal denoising using non-local wavelet transform domain filtering. IET Signal Process..

[27] Starck J.L., Fadili J., Murtagh F. (2007). The undecimated wavelet decomposition and its reconstruction. IEEE Trans Image Process.

[28] Jambukia S.H., Dabhi V.K., Prajapati H.B. Classification of ECG signals using machine learning techniques: A survey. Proceedings of the 2015 International Conference on Advances in Computer Engineering and Applications.

[29] Maknickas V., Maknickas A. Atrial Fibrillation Classification Using QRS Complex Features and LSTM. Proceedings of the 2017 Computing in Cardiology Conference (CinC).

[30] Zhang Z., Xu Y., Yang J., Li X., Zhang D. (2015). A Survey of Sparse Representation: Algorithms and Applications. IEEE Access.

[31] Azimi-Sadjadi M.R., Kopacz J., Klausner N. K-SVD dictionary learning using a fast OMP with applications. Proceedings of the 2014 IEEE International Conference on Image Processing (ICIP).

[32] Walsh N.P., Alba B.M., Bose B., Gross C.A., Sauer R.T. (2003). OMP peptide signals initiate the envelope-stress response by activating DegS protease via relief of inhibition mediated by its PDZ domain. Cell.

[33] Pati Y.C., Rezaiifar R., Krishnaprasad P.S. Orthogonal matching pursuit: Recursive function approximation with applications to wavelet decomposition. Proceedings of the 27th Asilomar Conference on Signals, Systems and Computers.

[34] Gacek A., Pedrycz W. (2011). ECG Signal Processing, Classification and Interpretation: A Comprehensive Framework of Computational Intelligence.

[35] Wang J., She M., Nahavandi S., Kouzani A. (2013). Human Identification from ECG Signals Via Sparse Representation of Local Segments. IEEE Signal Process. Lett..

[36] Lee W., Kim S., Kim D. (2018). Individual Biometric Identification Using Multi-Cycle Electrocardiographic Waveform Patterns. Sensors.

[37] Dong X., Si W., Huang W. (2018). ECG-based identity recognition via deterministic learning. Biotechnol. Biotechnol. Equip..

[38] Pelc M., Khoma Y., Khoma V. (2019). ECG Signal as Robust and Reliable Biometric Marker: Datasets and Algorithms Comparison. Sensors.

[39] Dar M.N., Akram M.U., Shaukat A., Khan M.A. ECG Based Biometric Identification for Population with Normal and Cardiac Anomalies Using Hybrid HRV and DWT Features. Proceedings of the 2015 5th International Conference on It Convergence and Security (Icitcs).

[40] Meltzer D., Luengo D. (2023). Efficient Clustering-Based electrocardiographic biometric identification. Expert Syst. Appl..

[41] Kim B.H., Pyun J.Y. (2020). ECG Identification for Personal Authentication Using LSTM-Based Deep Recurrent Neural Networks. Sensors.

[42] Fatimah B., Singh P., Singhal A., Pachori R.B. (2022). Biometric Identification from ECG Signals Using Fourier Decomposition and Machine Learning. IEEE Trans. Instrum. Meas..

